# PD23 - Decision points for boiled egg challenges in Greek children with sensitisation to egg proteins

**DOI:** 10.1186/2045-7022-4-S1-P23

**Published:** 2014-02-28

**Authors:** Marianna Tziotou, Maria Psomiadou, Anna Pananaki, Anastasios P Konstantinopoulos, Dimitrios Koutsalitis, Dimitrios Karantoumanis, Anastasia Karamouza, Eirini Roumpedaki, Stavroula Giavi, Emmanouil Manousakis, Nikolaos Douladiris, Nikolaos G Papadopoulos

**Affiliations:** 1 Allergy Department, 2nd Pediatric Clinic, University of Athens, Athens, Greece

## Introduction

Decision points based on food-specific IgE antibody concentrations have been proposed and are used in the clinic, in order to predict reactivity to foods and reduce the number of high risk food challenges. In the process of screening for eligibility in a study, we started carrying out open food challenges to boiled egg in children with sensitization to egg proteins, independent of their skin reactivity and specific IgE concentrations. In several occasions, the expected cut-off points were not confirmed. Furthermore, cut-off points have been evaluated in children with clinical indications of allergy and not in those who are only sensitized. Therefore, we hypothesized that decision points for this population may need to be reevaluated.

## Patients and methods

We retrospectively reviewed the medical records of children who underwent open challenges with boiled egg between October 1^st^ 2012 and April 30^th^ 2013 in our Unit. We included children who only had sensitization to egg and had never consumed egg in any form before. The patients’ characteristics (age, sex, medical history) and the results of the skin prick tests and sIgEs (CAP-FEIA) were collected and analyzed.

## Results

Sixteen children (9 boys,56.3%) were included. The mean age was 18 months (range 13-33 months).Thirteen (81.3%) suffered from AD, and 12 (75%) had another food allergy as well. The range of sIgE was 0.39-100KU/L for egg white (f1) and 0.10-50.5KU/L for egg yolk (f75).The range of SPT was 4-12mm for egg white, 0-5.5mm for egg yolk and 0-7mm for ovomucoide. Only 2 challenges (12.5%) were positive (f1=2.25, f75=0.35 and f1=3.84, f75=1.07 respectively).The 1-sided 95% confidence interval for G-mean of sIgEs and wheal diameters was calculated for the children with negative challenge: f1(-∞-16.83KU/L) p>0.1, f75 (-∞-4.97KU/L) p>0.1, egg white diameter (-∞-7.56mm) p<0.05, egg yolk diameter (-∞-3.77mm) p<0.1 and ovomucoide diameter (-∞-3.12mm) p<0.05.

## Conclusion

Children with IgE sensitization to egg, without previous egg consumption or reported reactions, have a considerable chance (12.5%) of being egg allergic. However, decision points reported for egg-allergic children do not appear to be valid in this population and need to be revised.

